# Long Non-Coding RNAs in Neuronal Aging

**DOI:** 10.3390/ncrna4020012

**Published:** 2018-04-18

**Authors:** Diana Pereira Fernandes, Mainá Bitar, Frank M. J. Jacobs, Guy Barry

**Affiliations:** 1 University of Amsterdam, Swammerdam Institute for Life Sciences (SILS), 1098XH Amsterdam, The Netherlands; d.s.pereirafernandes@uva.nl; 2 QIMR Berghofer Medical Research Institute, Herston, QLD, Brisbane, Australia; Maina.Bitar@qimrberghofer.edu.au

**Keywords:** lncRNAs, aging, cognition, neurogenesis, age-related diseases, neurodegeneration, antisense transcript, nuclear architecture, transposable element

## Abstract

The expansion of long non-coding RNAs (lncRNAs) in organismal genomes has been associated with the emergence of sophisticated regulatory networks that may have contributed to more complex neuronal processes, such as higher-order cognition. In line with the important roles of lncRNAs in the normal functioning of the human brain, dysregulation of lncRNA expression has been implicated in aging and age-related neurodegenerative disorders. In this paper, we discuss the function and expression of known neuronal-associated lncRNAs, their impact on epigenetic changes, the contribution of transposable elements to lncRNA expression, and the implication of lncRNAs in maintaining the 3D nuclear architecture in neurons. Moreover, we discuss how the complex molecular processes that are orchestrated by lncRNAs in the aged brain may contribute to neuronal pathogenesis by promoting protein aggregation and neurodegeneration. Finally, this review explores the possibility that age-related disturbances of lncRNA expression change the genomic and epigenetic regulatory landscape of neurons, which may affect neuronal processes such as neurogenesis and synaptic plasticity.

## 1. Introduction

Aging of the human brain often leads to cognitive decline [1], reduced neurogenesis [2] and neurodegeneration [3]. Such neuronal vulnerability makes aging the primary risk factor for neurodegenerative diseases. Alterations in the aging brain include changes in the epigenetics [4,5] and transcription [6] of both coding and non-coding regions of the genome.

Among non-coding transcripts, long non-coding RNAs (lncRNAs) have recently emerged as key regulators of the molecular processes that underlie age-associated phenotypes [7,8]. lncRNAs are transcripts that are longer than 200 nucleotides in length with virtually no protein-coding capacity [9]. These transcripts are mostly uniquely expressed in cell types—both spatially and temporally—and are particularly enriched in the brain, where they play functional roles in neuroplasticity, cognition, and differentiation of neural stem cells [10,11]. Additionally, lncRNAs are known to orchestrate epigenetic processes through their interactions with epigenetic machinery [12]. Interestingly, differential expression of lncRNAs has been described not only in healthy aging [13,14], but also in developmental and neurodegenerative diseases [15], raising the question of whether lncRNAs play a role in the aging of the human brain.

This review proposes ways by which lncRNAs may contribute to neural aging and how their functions can be altered across the human lifespan. We discuss that antisense (AS) lncRNAs can regulate pathological protein aggregation and that subnuclear compartment specific (SCS) lncRNAs can regulate neuronal splicing, transcription, and sponging of ion channels in aging. Other pre- and post-transcriptional regulatory roles performed by lncRNAs are also discussed in the context of cognition, neurogenesis, and neurodegeneration in aging, including the possible influence of lncRNAs on the maintenance of the 3D nuclear architecture.

## 2. Long Non-coding RNAs in Adult Neurogenesis: Implications for Aging

Neurogenesis is the process by which new functional neurons are generated from neural stem cells (NSCs) throughout life. In the mammalian adult brain, NSCs persist in the subgranular zone of the dentate gyrus of the hippocampus and in the subventricular zone (SVZ) of the lateral ventricles [16,17]. The discovery of neurogenesis in the adult mammalian brain, and its widespread decline throughout aging [2,18,19,20,21], suggest that the loss of the capacity for neurogenesis is a possible cause of aging [2,22]. While the origin of this neurogenesis decline with age is not yet clear, studies performed on rodents show both a significant reduction in the numbers of NSCs [18,19] and their proliferative potential [20,21]. These studies are corroborated by the finding that the hippocampus of a human brain displays a decline in the turnover of both neuronal and non-neuronal cells during aging [23]. The persistence of neurogenesis in the adult brain is believed to attenuate age-related phenotypes in two ways: (1) a decreased neuronal turnover would compromise neuron replacement, which is required for repair mechanisms that are triggered by brain injury or age-related diseases [2] and (2) reduced hippocampal neurogenesis may be important in the age-related loss of cognitive ability, since newly generated neurons could enhance neuronal plasticity, learning, and memory. Indeed, a study by Drapeu et al., 2003, showed that the extent of memory dysfunction in aged rats is quantitatively related to their deficit in hippocampal neurogenesis [22].

Although lncRNAs have been found to play crucial roles in the developing mammalian brain, little is known about their function in post-natal and adult neurogenesis. It is conceivable that the same lncRNA pool that regulates NSC behavior in the embryonic stage is equally important in adults, since neuronal development pathways are highly conserved among embryonic, early post-natal, and adult neurogenesis [16]. A study conducted by Barry et al., 2015 [13], reported that lncRNAs previously linked to embryonic neurogenesis (e.g., *MALAT1; BCYRN1; MIAT; SOX2-OT, TUG1,* and *RMST*) [11] were also expressed in the SVZ of the human adult brain [13]. *Dlx1as* [24], *Six3os* [24] and *Pnky* [25] are amongst the first lncRNAs whose functionality has been confirmed in adult mouse neurogenesis. Although several other ncRNAs were found to be specifically expressed or enriched in the neurogenic regions of the brain, their exact function remains unknown [26,27].

**Figure 1:**
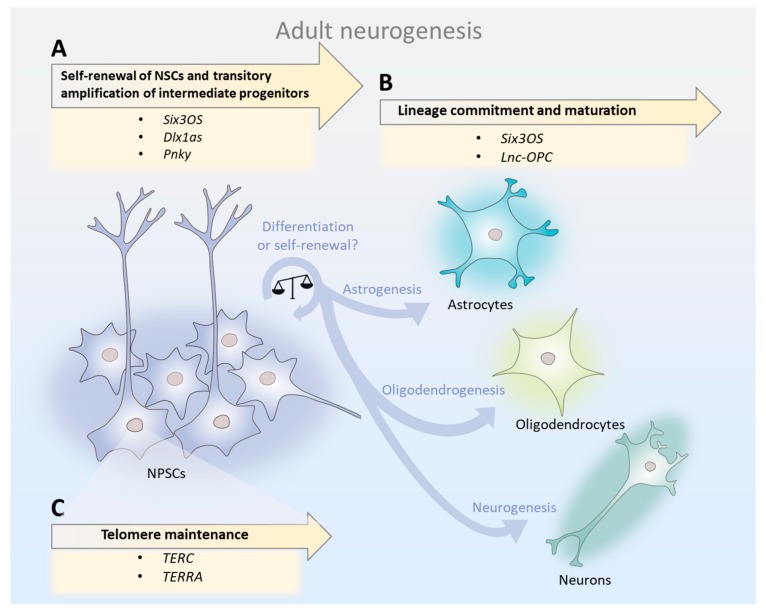
Long non-coding RNAs (lncRNAs) orchestrate temporally and spatially precise gene regulatory networks involved in the course of adult neurogenesis. (**A**) lncRNAs influence neural stem cells’ (NSCs) proliferation, expansion of transit-amplifying cells and differentiation into neuroblasts. In aging, mild alterations in lncRNA expression in the subventricular zone (SVZ) may compromise these processes, thus accounting for a decline in neurogenesis. (**B**) lncRNAs participate in lineage commitment and cell maturation. In the aged SVZ, the balance between lncRNAs involved in glial and neuronal fate specification may determine the cell fate of NSCs, leading to alterations in long-term neuronal/glial turnover. (**C**) lncRNAs are critical for telomere homeostasis. It is likely that an interplay between the telomeric lncRNAs *TERRA* (telomeric repeat containing RNA) and *TERC* (telomerase RNA component) regulates telomerase activity and the survival of NSCs during aging.

Taken together, studies on the developing and adult mammalian brain suggest that the relative abundance of individual lncRNAs in the total NSC pool may determine the course of neurogenesis. lncRNAs are key modulators of NSC maintenance, lineage commitment/differentiation, and telomere maintenance (Figure 1). Therefore, since lncRNAs orchestrate temporally and spatially precise gene regulatory networks that are involved in neurogenesis, mild alterations in their expression in the aged SVZ may account for the neurogenesis decline.

### 2.1. lncRNAs in Neural Stem Cells: Self-Renewal, Amplification of Intermediate Progenitors, and Generation of Neuroblasts

In adult neurogenesis, activated NSCs give rise to transit-amplifying cells, which in turn generate neuroblasts [16]. lncRNAs play major stage-dependent roles, influencing not only the transition from one stage to the other, but also the number and type of cells generated in each stage. For instance, knockdown of the lncRNA *Six3os* in the SVZs of adult mice NSCs resulted in a two-fold decrease in Tuj1-positive cells (neuronal marker) and increased GFAP-positive cells (activated NSC marker) [24]. Moreover, *Dlx1as* knockdown in adult murine SVZ of NSCs caused a three-fold decrease in Tuj1-positive neuroblasts, an increase of nearly 60% in GFAP-positive cells, and a decrease in the expression of *Dlx1* and *Dlx2*, which are two transcription factors that play major roles in neuronal development [24]. These results demonstrate that *Six3os* and *Dlx1as* are important for the amplification of intermediate progenitors and neuroblast generation. In contrast, *Pnky* knockdown in post-natal NSCs potentiates neuronal lineage commitment and expands the transit-amplifying cell population, increasing neuron production by several-fold [25]. Therefore, *Pnky* is important for NSC maintenance.

It is likely that subtle alterations in the levels of these lncRNAs have dramatic effects on destabilizing the dynamic balance that is established between NSC proliferation, intermediate amplification, and differentiation into neuroblasts, potentially leading to impaired neurogenesis in aging. Importantly, *DLX1AS* expression in the SVZ is not altered during aging [13]. However, a recent study showed that the expression of the lncRNAs *MALAT1, GOMAFU, NEAT1,* and *TUG1* in the human SVZ significantly increases with age [13]. This may be related to their functions in non-neuronal cells, where they control cell-cycle [28] and senescence [29].

### 2.2. lncRNAs in Cell Lineage Commitment: Shifting from Neurogenesis to Oligodendrogenesis in Aging?

In addition to the neuronal cell lineage, NSCs also generate glial lineages, which give rise to astrocytes, oligodendrocytes, and ependymal cells [16]. Many lncRNAs exhibit dynamic expression patterns during neuronal–glial fate specification, suggesting roles in cell lineage commitment [30]. Transcript knockdown of *Six3os* lncRNA resulted in three-fold fewer cells expressing the oligodendrocyte marker OLIG2 [24], suggesting its involvement in the gliogenic specification of NSCs. Furthermore, long non-coding RNA-oligodendrocyte precursor cell *(lnc-OPC)* depletion resulted in a significant decrease in the expression of oligodendrocyte precursor cell (OPC) markers (MBP, PLP1, and CNP) and O4^+^ (oligodendrocyte surface marker), demonstrating that *lnc-OPC* plays a role in oligodendrogenesis [30].

lncRNAs may also direct a neurogenic fate in NSCs. For example, knockdown of the lncRNA *RMST* blocks neuronal differentiation and is required for the binding of SOX2 to promoter regions of neurogenic transcription factors [31]. Interestingly, a study conducted by Capilla-Gonzalez et al., 2013 reported that while the production of new neurons decreases during aging, the generation of oligodendroglial cells is not compromised in the murine SVZ [32]. The authors hypothesize that the preservation of oligodendrogenesis may be crucial for myelin maintenance in the aged brain [2].

### 2.3. Telomeric lncRNAs: Lying at the Root of Aged NSCs Survival?

Telomeres are repetitive DNA elements that cap the ends of chromosomes and protect their integrity. Throughout life, telomeres of somatic cells shorten at every round of DNA replication until the progressive and cumulative loss of telomere sequences ultimately triggers cellular senescence [33]. For this reason, telomere attrition is believed to be one of the main processes that determine the lifespan of somatic cells and organism aging. Several studies have demonstrated that aging can be delayed by telomerase activation and, accordingly, that pathological telomere dysfunction accelerates aging [34]. In germline and adult stem cells, telomere shortening can be countered by de novo addition of telomeric repeats by the TERT (telomerase reverse transcriptase) enzyme. In the adult mouse brain, telomerase activity is specific to NSCs isolated from the adult SVZ and hippocampus [35]. TERT expression is downregulated throughout aging in the SVZ of mice, leading to telomere shortening and strikingly disrupting neurogenesis and neuritogenesis [36]. Accumulating evidence suggests that telomere shortening is also an important cause of stem cell decline with aging in many other tissues [37,38]. Unsurprisingly, NSC functionality is highly dependent on telomere dynamics [36,39].

lncRNAs play key roles in telomere dynamics in stem cells. The lncRNA *TERC* (telomerase RNA component) forms a ribonucleoprotein complex with TERT, acting as a scaffold that brings the protein subunits of telomerase together, but also serves as a template for the synthesis of new telomeric repeats [40]. Both TERT and *TERC* are essential for telomere maintenance and elongation, as shown by their respective knockout mice models—which display short telomeres, instability, and premature aging [41,42]. Importantly, the lncRNA *TERC* is the limiting factor for telomerase activity as *TERT* heterozygote mice show no defects in telomere elongation, while *TERC* homozygotes do [42]. This finding suggests that the expression of the lncRNA *TERC* is not only crucial for telomerase activity, but also modulates it to promote and maintain telomere length. Interestingly, Klapper et al., 2001, observed that the temporal pattern of telomerase activity does not reflect the observed decrease in TERT transcript levels throughout pre- and post-natal neuronal development in mice [43]. Moreover, those same patterns of change occurred in association with decreased cell proliferation, differentiation, and natural cell death during early life neurogenesis. The authors propose a model in which the balance between TERT and *TERC* regulates neurogenesis, with high levels of TERT and *TERC* being responsible for NSC proliferation, low levels of *TERC* and high levels of TERT inducing differentiation, and low levels of both lncRNAs resulting in cell death. While the relative abundance of *TERC* and TERT in adult and aged NSCs remains unknown, this theory raises the question of whether a dysfunctional balance between *TERC* and TERT may trigger processes observed in aged NSCs, such as telomere de-protection (ultimately leading to senescence) or compromised cellular viability (leading to apoptosis).

Additionally, lncRNAs named *TERRA*s (telomeric repeat containing RNAs) are also key regulators of telomere dynamics. *TERRA* molecules are transcribed from the subtelomeric region of chromosomes and can be actively displaced to chromosome ends, the nucleosome [44,45], or the exterior of the cell, where they exist as components of inflammatory exosomes [46]. At chromosomal ends, *TERRA* transcripts can base-pair with complementary DNA, forming RNA:DNA hybrid structures that regulate telomere length [47,48]. In cells that display telomerase activity, the role of *TERRA* transcripts remains unclear. On one hand, in vitro experiments using *TERRA*-mimicking oligonucleotides suggest that *TERRA*s inhibit telomerase activity by directly binding to both TERT and *TERC* [49]. On the other hand, in yeast, *TERRAs* transcription is induced at short telomeres and form *TERRA*-telomerase RNA clusters in the early S phase. These are later recruited to short telomeres from which the *TERRA*s originated, triggering telomere elongation [45]. Interestingly, telomerase preferentially elongates short telomeres during the late S phase, a time point where *TERRAs* levels decline at telomeres [50]. Therefore, it appears that the dynamic balance of *TERRA* molecules throughout the cell cycle is a crucial factor that sustains and regulates telomere length [44,51]. Besides directly modulating telomerase activity, *TERRAs* are also proposed to regulate telomere length via heterochromatin formation at chromosome ends [52], capping of telomeres [53,54,55], and cellular differentiation [56]. Moreover, a recent study demonstrated that *TERRA* subtelomeric region knockouts in three human cell lines (HeLa, HCT116, and U2OS) are often lethal and lead to a dramatic loss of telomere sequences and a massive induction of the DNA damage response [55].

Taken together, these studies suggest that *TERRA*s are involved in multiple functions that mediate genomic instability, cell survival, and cellular senescence. As these functions are extensively affected through life, a role for *TERRAs* in aging can be reasonably inferred (e.g., [8,57]). Interestingly, the repression of general subtelomeric transcription by transiently activating mitochondrial reactive oxygen species in yeast was found to extend this organism’s lifespan [58]. In line with these findings, *TERRA* expression levels have been inversely correlated with telomere length [59,60], which implies that *TERRA*s are upregulated during aging. Human and murine induced pluripotent stem cells (iPSCs) have constantly elevated levels of *TERRA* transcripts [61,62]. Additionally, *TERRA* overexpression is found in proliferating progenitor cells in the developing mouse brain that exhibits *TERRA* foci [60]. This expression pattern supports the idea that high *TERRA* expression is coupled with cellular proliferation in progenitor cells. Therefore, *TERRA* expression in the presence of reduced telomerase activity—which is typical of aging—could lead to NSC cellular arrest and eventual senescence. The state of NSCs in aging is likely dependent on the interplay that is established between *TERC, TERRA,* and TERT. A shift in the abundance or activity of one of these molecules, both overall and in a cell-cycle-stage specific manner, could be implicated in the progression of aging.

## 3. lncRNAs in Cognitive Decline

Aging is associated with impairments in cognitive functions, including loss of memory and synaptic plasticity, and altered activation of the prefrontal cortex and hippocampus [1,63]. Cognitive decline seems to be independent of neuronal loss and may rather be a direct consequence of the alterations in synaptic connectivity [1]. The current understanding of learning, memory, and cognition is that neuronal activity is responsible for continuous changes in the synaptic connections that are established between neurons [1,64,65]. Modification of synaptic strength in the adult circuitry occurs through cellular mechanisms such as long-term potentiation (LTP) and long-term depression (LTD) [64,65]. Underlying these processes is a series of molecular events such as the activation, synthesis, and relocation of certain neurotransmitter receptors [64,65] and ion channels [65]. Likewise, local protein synthesis in dendrites [64,65,66] is regulated in response to neuronal activity, and is maintained by an asymmetric distribution of mRNAs and a control of their transcription, location, transport, and splicing [66]. The roles for lncRNAs in modulating the molecular processes on the basis of synaptic plasticity throughout life are just starting to emerge [67].

### 3.1. The Synaptic Coding/Non-Coding Interactome: Emerging Functions for lncRNAs on Synaptic Plasticity-Associated Genes, Transcripts, and Proteins

**Figure 2:**
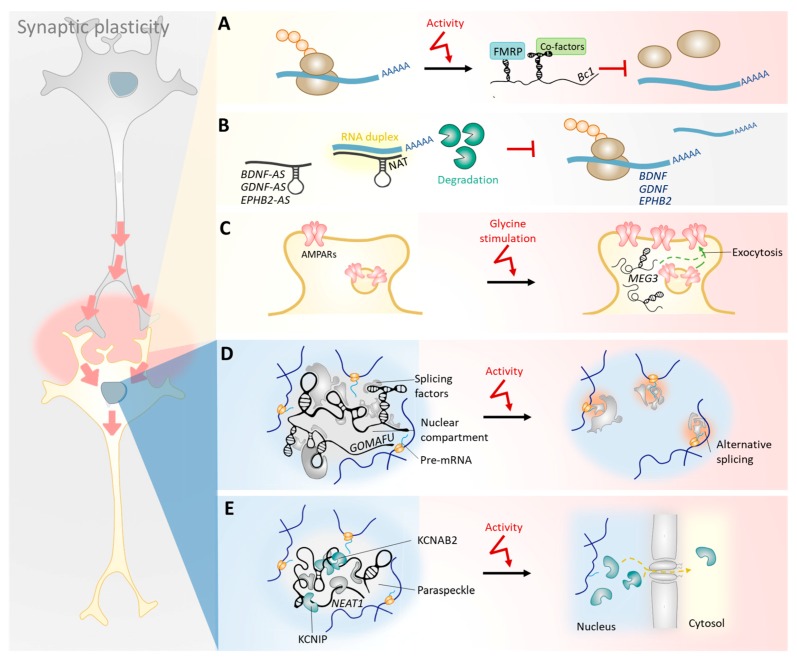
Mechanisms underlying the lncRNA function in synaptic plasticity. lncRNAs respond to neuronal activity and not only regulate the expression of genes involved in neurite outgrowth, but also modulate ion channel stoichiometry, thereby altering the synaptic connectivity and excitatory properties of a neuron. (**A**) Upon neuronal activity, certain lncRNAs (e.g., *BC200*, *Bc1*) are transcribed and transported to dendrites, where they regulate local protein translation rates. (**B**) The stability of protein-coding transcripts involved in synaptic plasticity may be controlled by their antisense pairs (e.g., *BDNF-AS*, *GDNF-AS*, *EPHB2-AS*) in dendrites. (**C**) In response to glycine stimulation, some cytosolic lncRNAs (e.g., *MEG3*) regulate the trafficking of AMPA receptors to the plasma membrane, modifying the excitatory landscape of a neuron. (**D**) Nuclear-retained lncRNAs (e.g., *GOMAFU*) control the activity-dependent release of splicing factors to regulate gene expression and splice variant distributions that influence dendritic growth, morphology, and function. (**E**) Nuclear lncRNAs (e.g., *NEAT1*) also control the activity-dependent transcription and nucleus-to-cytosol shuttling of ion channel subunits, altering the excitatory properties of a neuron. FMRP, fragile X mental retardation protein.

The regulation of synaptic plasticity by lncRNAs is a complex task that relies not only on their selective transport [68] to the dendrites of mature neurons [67,69,70], but also on the direct modulation of their expression levels by neuronal activity [71,72]. Thus, LTP studies in the dentate gyrus of living rats revealed dynamic expression profiles of lncRNAs that were highly correlated with synaptic plasticity-associated protein-coding genes [73]. Growing evidence suggests that, once in specific locations and at appropriate physiological levels, lncRNAs regulate the expression of such genes at both pre- and post-transcriptional levels, as discussed in more detail below (Figure 2). For instance, certain dendritic lncRNAs regulate local protein translation rates and the stability of protein-coding transcripts [74,75,76,77,78]. Furthermore, other nuclear-retained lncRNAs regulate the transcription of genes involved in the functioning of synapses, their splicing, and the nucleus-to-cytosol shuttling of ion channel subunits [72,79,80,81]. These lncRNA-mediated regulatory mechanisms may be involved in dynamic alterations in the synaptic connectivity and excitatory properties of a neuron.

### 3.2. lncRNAs Regulate Local Protein Translation Rates in Synapses

The regulation of local protein translation is partially carried out by the lncRNAs *Bc1* and *BC200*, expressed in rodent and primate brains, respectively. In response to neuronal activity, *Bc1* and *BC200* are upregulated [82] and transported to dendrites [83], where they act as a scaffold that interacts with the translational machineries and represses local translation in synapses [74,75,76]. *Bc1* gene knockout in mice results in neuronal hyperexcitability, convulsive seizures [84], anxiety, and exploratory behavioral defects [85]. *Bc1* and *BC200* activity is, therefore, crucial for normal neuronal activity and behavior. Importantly, *BC200* levels in cortical areas are reduced by >60% between the ages of 49 and 86 years in healthy human individuals [86], raising the question of whether its expression changes may contribute to age-related cognitive decline.

### 3.3. Antisense lncRNAs Locally Regulate the Stability of Protein-Coding mRNAs Involved in Synaptic Plasticity

Among the classes of mRNAs expressed near synapses, natural sense/antisense transcript pairs are commonly found in the adult mouse forebrain [67]. Natural AS lncRNAs are transcripts that overlap—at least partially—with the mRNA of the coding gene. Given their significant sequence overlap, sense/antisense transcripts usually hybridize in an RNA duplex when in close proximity. This RNA structure regulates the stability of the coding mRNA and thus its protein level [87]. Importantly, some synaptic AS lncRNAs were found to downregulate the expression levels of proteins involved in neurite elaboration, such as BDNF [77,78], GDNF [77], and EPHB2 [77]. The core potassium channel subunit KCNA2 is also regulated by an AS lncRNA in response to peripheral nerve injury [88].

While some groundbreaking studies showed that antisense regulation of gene expression in synapses plays important roles in neuronal plasticity, its impact in aging remains unknown. Interestingly, in *Aplysia* sp., the regulation of sensorin (SRN), a gene involved in learning and long-term memory with no homologue in mammals, is performed by an AS lncRNA with an expression that is reduced in aging [89]. This study also reported that the distribution of SRN and *SRN-AS* within a neuron becomes asymmetric in aging. Hence, we hypothesize that a decreased generation of AS lncRNAs that are capable of dynamic gene regulation or the aberrant spatial distribution of sense/antisense transcripts in an aged neuron, might underlie local protein level defects and, consequently, synaptic plasticity deficiency.

### 3.4. Nuclear lncRNAs Dynamically Regulate the Transcription and Splicing of Coding Transcripts Involved in Synaptic Plasticity

lncRNAs also mediate the splicing of pre-mRNAs with retained introns. It has become increasingly clear that a dynamic regulation of splicing in the nervous system is critical for neuronal development and for the establishment and maintenance of neuronal networks [90]. A recent study by Traunmülle et al., 2016 was the first to elucidate the impact of splicing programs specifically in synapses [91]. The disruption of splicing patterns by interfering with a single RNA-binding protein, SLM2, resulted in defects in trans-synaptic protein complexes, impaired glutamatergic transmission, and synaptic plasticity [91]. Synaptic splicing programs are thus being regarded as creators of different, highly specific types of synapses, and thereby as mediators of neuronal plasticity. Interestingly, some lncRNAs are retained in the nucleus and predominantly localize to specific nuclear sub-compartments that are enriched in pre-mRNA splicing and processing factors, where they work as RNA-binding proteins (as discussed below).

As an example of lncRNAs located in subnuclear compartments, the lncRNA *MALAT1* is specifically enriched in nuclear speckles, where it is proposed to act as a splicing factor sponge [92]. Accordingly, *MALAT1* was shown to modulate the recruitment of SR family pre-mRNA-splicing factors to an active transcription site of a reporter gene locus [79]. Surprisingly, the depletion of *MALAT1* in neuroblastoma cells affected not only the expression of genes involved in nuclear processes, but also that of genes in synapse function and dendrite development [79]. In line with this finding, *MALAT1* knockdown in cultured hippocampal neurons significantly decreased the synaptic density, whereas its overexpression resulted in increased synaptic density [79]. Hence, *MALAT1*, in a similar way to the RNA-binding protein, SLM2, modulates the expression and splicing of genes involved in synapse function and maintenance. While this mechanism is not fully understood, further insights from studies of the lncRNA *GOMAFU* help to draw a possible working model.

Like *MALAT1*, *GOMAFU* is retained in the nucleus and is localized in another specific nuclear compartment [93], where it binds to splicing factors [72,94]. Loss-of-function mutations of *GOMAFU* in human iPSC-derived neurons lead to alternative splicing patterns [72] in the synaptic plasticity-related genes *DISC1* [95,96,97,98], *ERBB4* [99,100], and *DRD2* [101,102]. Interestingly, upon depolarization of mouse primary cortical neurons and iPSC-derived neurons by KCl, *GOMAFU* transcript levels are downregulated while *MALAT1* levels remain unchanged [72]. This suggests that *GOMAFU* regulates plasticity-related, activity-dependent alternative splicing. Thus, the authors suggest a model in which *GOMAFU* acts as a splicing factor scaffold in the nuclear compartments of an inactivated neuron. Upon neuronal activation, *GOMAFU* expression is downregulated, allowing for the release of splicing factors into the nucleoplasm, where they can modulate the splicing of transcripts involved in synaptic and dendritic growth, morphology, and function [72]. Although this is still a speculative hypothesis, it is likely that *MALAT1* works in a similar way, but that its expression is regulated by stimuli other than the KCl-induced depolarization of neurons.

Taken together, these studies demonstrate that lncRNAs can act as RNA-binding proteins in specific subnuclear compartments and are dynamically regulated in response to neuronal activity, allowing for the splicing of genes involved in neuronal plasticity. Even though this mechanism remains poorly characterized, it would be very interesting to test these ideas in aging models in vivo.

### 3.5. Nuclear lncRNAs Regulate the Transcription and Nucleus-to-Cytosol Shuttling of Ion Channel Subunits in Response to Neuronal Activity

The lncRNA *NEAT1* is retained in the nucleus, where it aggregates into paraspeckles structures [103]. *NEAT1* expression level is dynamically regulated by neuronal activity and binds potassium channel-interacting proteins, including KCNAB2 and KCNIP [80]. The modulation of the stoichiometry of potassium channel protein subunits [104] and other ion channels [105] regulates neuronal excitability and thereby neuronal plasticity. Similarly, the shuttling of ion channel components from the nucleus to the cytosol may fine-tune the activity of ion channels, since these proteins can now interact with membrane channels. *NEAT1* appears to be particularly important in this process as its transient downregulation in response to neuronal activity induces the release of potassium channel proteins, such as KCNAB2, from the nucleus into the cytosol [80]. Once in the cytosol, KCNAB2 is able to fine-tune the excitatory response, since knockdown of *NEAT1* transcript induces a neuronal hyper-potentiation phenotype in iPSC-derived human cortical neurons [80]. *NEAT1* is also involved in the transcriptional regulation of ion channel components as its knockdown in activated neurons drives a significant increase in ion channel gene expression [80]. Interestingly, the modulation of intrinsic neuronal excitability is a process that is severely affected in normal aging, and which may account for the learning impairment observed in normal aging subjects [106]. Additionally, dysregulation of *NEAT1* activity may be involved in this phenotype.

### 3.6. lncRNAs Co-Expressed in the Nucleus and Cytoplasm Regulate the Trafficking of AMPA Receptors to the Plasma Membrane in Response to Glycine Stimulation

A recent study reported that the expression of a lncRNA cluster—consisting of *MEG3*, *MEG8*, *MEG9,* and *RTL1-AS*—in primary cortical neurons following glycine stimulation occurred in an *N*-Methyl-d-aspartate receptor (NMDAR)-dependent manner [81]. *MEG3* knockdown blocked the glycine-induced increase of the GluA1 subunit of AMPA (α-amino-3-hydroxy-5-methyl-4-isoxazolepropionic acid) receptors at the plasma membrane by regulating its trafficking, which shows that *MEG3* may have a role in the regulation of LTP [81].

### 3.7. Loss of Nuclear and Cytoskeleton Integrity as an Underlying Cause of Aging Synapses

While we have focused on alterations in the expression of lncRNAs that directly modulate the synaptic response, factors intrinsic to the cellular organization of an aged neuron may also modify lncRNA-associated synaptic activity. Nuclear and cytoplasmic [107] cellular organization of neurons contribute to modulate cell-autonomous processes, such as cytoskeletal protein transport [108,109] and nucleocytoplasmic compartmentalization [110,111]. The organization of the cytoskeleton in neurons suffers widespread alterations throughout aging [107,112] that impair protein transport [108], potentially compromising the transport of lncRNAs to dendrites and dysregulating the local dendritic processes that they orchestrate. The regulation of synaptic connectivity also depends on the selective exportation of a subset of lncRNAs from the nucleus to the cytoplasm. Since there is loss of cellular compartmentalization in aged neurons resulting from age-dependent nuclear pore deterioration [110], this transport may be compromised, and lncRNAs such as *Bc1* and *BC200* may leak into the cytoplasm, counteracting their neuronal activity-dependent regulation. The loss of cell nucleocytoplasmic compartmentation also poses a threat for nuclear lncRNAs. Firstly, cytoplasmic proteins and RNAs that leak into the nucleoplasm can compete with endogenous factors for binding sites in scaffolding lncRNAs, interfering with the specificity of their regulation. Secondly, the activity-dependent binding of ion channel-interacting proteins in the nucleus may also be compromised by their leakage into the cytoplasm. This would most likely give rise to hyperexcitability phenotypes, which have been found in aged CA3 pyramidal neurons [113]. Additionally, the nuclear lncRNAs that are discussed here exist in very specific subnuclear compartments, where they bind specific molecules and thus orchestrate the regulation of different, non-overlapping synaptic processes. A question that arises is whether genomic instability, re-organization of DNA architecture, and increased transcriptional noise in aging might cause non-specific binding of proteins and transcripts to different lncRNAs in different subnuclear domains. The abundance and spatial distribution of nuclear substructures in aging also remain elusive.

## 4. lncRNA-Mediated Processes in the Pathogenesis of Neurodegenerative Disorders

The transcriptomes of mammalian brains show widespread changes during aging [6]. In addition to changes in transcript expression and the usage of alternative isoforms and promoters of protein-coding genes [114,115,116,117], quantitative and qualitative changes in the non-coding transcriptome also occur with age [115,118]. Epigenetic alterations inherent to the aging process, such as altered patterns of histone post-translational modifications and DNA methylation, have been considered as the basis for an aged transcriptome [4,8]. Extensive epigenetic rearrangements result in chromatin remodeling, leading to alterations in the local accessibility of the genetic material, and thus affecting gene expression [4].

**Figure 3:**
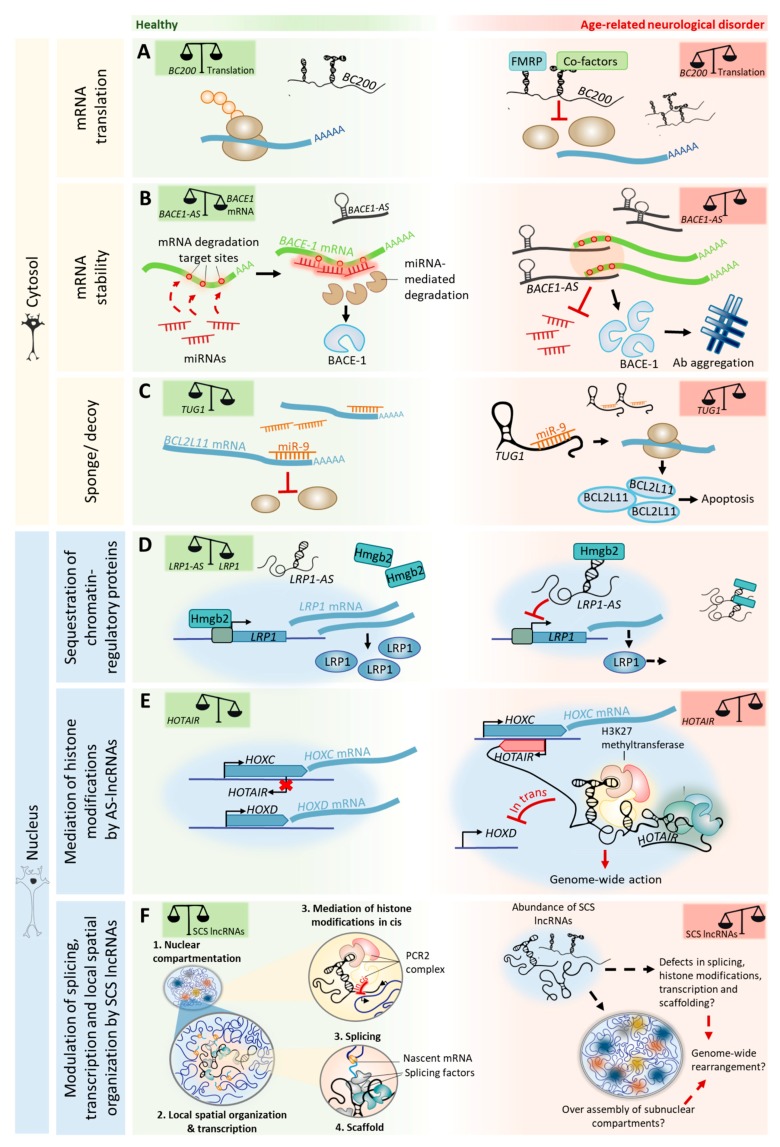
lncRNA-associated mechanisms in the healthy brain and in age-related neurodegenerative disorders. A shift in lncRNA abundance triggers alterations in the pre-transcriptional (blue columns) or post-transcriptional (yellow columns) regulation of neuronal genes, increasing susceptibility to disease and cognitive decline. (**A**) Certain lncRNAs inhibit or promote the translation of specific target mRNAs. The lncRNA *BC200* represses translation by recruiting translational machinery. (**B**) Natural sense/antisense transcripts hybridize and regulate the stability of coding (sense) mRNAs involved in synaptic plasticity and neurodegeneration. Hybridization of *BACE1-AS* with *BACE1* mRNA inhibits *BACE1* transcript miRNA-mediated decay, ultimately leading to overexpression of BACE1 in the brains of Alzheimer’s disease patients and accounting for amyloid-beta (Aβ) pathological aggregation. (**C**) In the cytosol, lncRNAs can also act as a sponge for miRNAs in order to sequester them and prevent them from binding to target transcripts. The lncRNA *TUG1* traps miRNA-9, preventing it from binding to the 3’UTR of *BCL2L11* mRNA, where it inhibits *BCL2L11* translation. As a result, the pro-apoptotic factor BCL2L11 is overexpressed and induces apoptosis in ischemia. (**D**) In the nucleus, lncRNAs can also act as a sponge to ubiquitous chromatin-regulatory proteins, fine-tuning their activity. The lncRNA *LRP1-AS* binds to HMGB2, preventing it from enhancing *LRP1* transcription in cis, and thereby reducing its expression. (**E**) Nuclear lncRNAs have affinity for chromatin-regulatory proteins, allowing for the assembly of complexes that base new histone modifications in cis or trans. The lncRNA HOTAIR silences the HOXD locus in trans by recruiting Polycomb proteins. Additionally, it can interact with multiple regulatory complexes simultaneously, having a genome-wide effect. (**F**) Subnuclear compartment specific (SCS) lncRNAs shape nuclear architecture by acting as epigenetic modulators of chromatin states in cis or trans, both by organizing the dynamic assembly and disassembly of subnuclear compartments in the periphery of active chromatin regions and by influencing the splicing or sponging of ion channels.

We suggest that changes in the expression levels of lncRNAs, caused by age-dependent epigenetic alterations, may impair or attempt to compensate for processes of adult neurogenesis, cognition, and neurodegeneration. The insights from age-related neurodegenerative disorders (Table 1) imply that the relative abundance of specific lncRNAs in a neuron in a given space and time determines the narrow range in which lncRNA-mediated processes are beneficial, before becoming pathogenic (Figure 3).

### 4.1. Roles for AS lncRNAs in Neuronal Aging and Disease

More than half of mammalian coding genes have complementary non-coding AS transcription [119]. AS lncRNAs have emerged as important regulators of gene expression, being able to influence a myriad of processes from epigenetic regulation to splicing, stability, and translation of coding mRNAs [120].

In age-related neurodegenerative disorders, the dysregulation of AS lncRNAs plays crucial roles in pathological protein aggregation. In Alzheimer’s disease (AD), AS lncRNAs contribute to amyloid-beta (Aβ) aggregation by modulating the expression and/or splicing of proteins involved in the generation and trafficking of Aβ. For example, the lncRNA *BACE1-AS* hybridizes to *BACE1* mRNA [121], a protein involved in Aβ processing, and inhibits its cytoplasmic miRNA-mediated decay. *BACE1-AS* is upregulated in the brains of AD patients, consequently leading to the overexpression of BACE1 and to an increase in Aβ generation [122] (Figure 3A). *SORL1-AS*, which is also upregulated in the brains of AD patients, induces the synthesis of pathogenic splicing isoforms of SORL1, which are associated with increased Aβ levels in cultured human neuronal cells [123]. Furthermore, the AS lncRNA *UCHL1-AS* targets *UCHL1* mRNA to heavy polysomes for translation, resulting in increased UCHL1 protein levels [124]. *UCHL1* is a Parkinson’s disease (PD) and AD risk gene that is believed to prevent pathological protein aggregation by promoting its (or its precursors) ubiquitination [125,126]. Interestingly, *UCHL1-AS* is downregulated in PD [127]. In the nucleus, antisense transcript expression can also control the transcription initiation of coding genes by sequestering chromatin-regulatory proteins. *LRP1-AS* directly binds to HMGB2, preventing it from enhancing *LRP1* transcription, thereby reducing its expression [128] (Figure 3D). LRP1 modulates the integrity of dendritic spines, synapses, and neuronal viability, and is also involved in Aβ deposition [129]. LRP1 expression is downregulated with aging [130], compromising neuronal survival in the brains of aged mice [129]. Interestingly, *LRP1-AS* is upregulated in AD [128], thereby the sequestration of transcriptional activators such as HMGB2 may account for LRP1 downregulation with age and in neurodegenerative disorders.

Besides regulating protein aggregation, cytoplasmic AS lncRNAs can also regulate the stability of transcripts from genes linked to neurodegenerative disorders and cognition (e.g., *PINK1* [131], *GDNF, EPHB2* [77], and *KCNA2* [88]). The binding of lncRNAs to protein-coding transcripts may affect their stability and either protect them from degradation (e.g., *PINK1-AS*) or cause their decay (e.g., *GDNF-AS*, *EPHB2-AS,* and *KCNA2-AS*). In the nucleus, besides sequestering chromatin regulatory molecules, AS expression may also recruit transcriptional repressors to chromatin-modifying complexes that are close to gene promoters, thus silencing gene expression. A recent study showed that 20% of lncRNAs (out of a cohort of 3300) often associate with the polycomb repressive complex 2 (PRC2), a histone methyltransferase that catalyzes repressive H3K27 methylation [132]. For example, *BDNF-AS* recruits PRC2 to the *BDNF* promoter, resulting in transcriptional repression [77], which possibly affects neurite elaboration in aging. The HOX transcript antisense RNA (*HOTAIR*) binds and targets PRC2 in trans to the *HOXD* cluster [133] and to multiple sites of the genome [134]. *HOTAIR* can also interact with multiple regulatory complexes simultaneously. *HOTAIR* binds both PRC2 and the LSD1/coREST/REST complex—which catalyzes H3K4 demethylation—into a single ribonucleic complex [135]. By functioning as a scaffold for selected chromatin-modifying enzymes, *HOTAIR* can specify the pattern of histone modifications on target genes (Figure 3E). AS lncRNA-mediated PRC2 repression of genes can also happen in cis. For example, the nascent lncRNA *ANRIL*—antisense to the *INK4* locus—silences the *CDKN2B–CDKN2A* locus by recruiting PRC2, which induces H3K27 methylation and long-term promoter DNA methylation in the locus [136,137]. Interestingly, *HOTAIR*, *ANRIL,* and their targets (e.g., *HOXD9*, *HOXD10,* and *CDKN2*) are included in the ~2000 genes that are differentially expressed across aging in all human tissues [138]. Importantly, these lncRNAs are also associated with age-related neurodegenerative disorders, as *HOTAIR* is overexpressed in a mouse model of PD [139] and single-nucleotide polymorphisms in the *CDKN2B–CDKN2A* locus have been associated with AD pathology [140]. Furthermore, *ANRIL* regulates the expression of CDKN2B that accumulates in neurofibrillary tangles and amyloid plaques in the brains of AD patients [141].

### 4.2. Transposonable Elements as a Source of lncRNAs in Aging

Transposonable elements (TEs) are repetitive DNA elements that account for nearly 50% of the human genome [142]. TEs are present at transcriptional start sites of a significant number of lncRNAs and control their transcriptional regulation, functioning as promoters [143]. In certain families of TEs, such as the HERVH family, the regulation of lncRNA expression evolved in a tissue- and developmental phase-dependent way, highlighting the functional sophistication of lncRNAs [143]. For instance, HERVH-driven lncRNAs are required for pluripotency in human embryonic stem cells [143]. TEs are also a major source of genomic instability, thus eukaryotic genomes have evolved epigenetic “defense” mechanisms that keep the majority of these elements under strong repression, silencing their expression and preventing mobility [144,145]. Interestingly, mounting evidence from studies in the senescence and aging of multiple organisms suggests that alterations in the activity of TEs-associated repressors [146,147], and the loss of heterochromatin [148,149], underlie TE activation in aging [150,151]. As a result, de-repressed TEs harbor the potential to modify lncRNA expression levels. Age-dependent de-repression of TEs may also induce de novo lncRNA transcription. This was exemplified in a study in which it was found that expression of de novo lncRNAs from *Alu* TEs during adult human stem cell aging promotes senescence, whereas knockdown of *Alu* lncRNAs reverses senescence [152].

### 4.3. Chromatin Remodeling and Nuclear Architecture in Aging: the (Big) Impact of lncRNAs

Higher level chromatin organization within the nuclear space is termed nuclear architecture [153]. Organization of the nuclear architecture influences DNA stability and gene expression patterns [147] and, thereby, defines cell identity [154]. Changes in the nuclear architecture are a hallmark of aging and result in genomic instability and transcriptional deregulation. In fact, premature aging syndromes, such as the Hutchinson–Gilford progeria syndrome, the Werner syndrome, and ataxia telangiectasia, share dramatic disturbances in the nuclear architecture of cells, and defects in diverse sets of genes that are involved in their maintenance [153]. lncRNAs play crucial roles in organizing the nuclear architecture of neurons by three different, yet complementary, processes: (1) lncRNAs function as epigenetic modulators of chromatin states [12]; (2) SCS lncRNAs change the nuclear architecture and alter chromatin repositioning [155]; and (3) the act of lncRNA transcription defines/affects the nuclear architecture [156]. Each of these processes are discussed in detail below:

(1) As previously mentioned, many lncRNAs bind to chromatin-modifying proteins and recruit their catalytic activity in cis or trans to specific gene loci, thereby modulating chromatin states and impacting gene expression [12]. Modulation of chromatin states occurs in several loci simultaneously and likely contributes to the overall nuclear architecture of the neuronal genome. Therefore, disturbances in the chromatin state of a single locus are probably sufficient to trigger genome-wide chromatin readjustments, not only because of the constrained nature of the human genome, but also because of its transcriptional output. This process is particularly relevant in loci that coordinate complex transcriptional programs. For instance, the *INK4* and *HOX* loci coordinate the expression of genes involved in cell cycle regulation and developmental patterning, respectively. These loci also contain the lncRNAs *ANRIL* and *HOTAIR*, respectively, which have altered expression in human tissues during aging [138]. While *ANRIL* and *HOTAIR* may function in cis for loci regulation, and are likely to play roles in post-mitotic neuronal processes, their dysregulation may also trigger the aberrant re-activation of cell cycle and developmental transcriptional programs—changes that are typically found in neuronal aging [157,158].
(2) lncRNAs influence the nuclear architecture directly by organizing the dynamic assembly and disassembly of subnuclear compartments in the periphery of active chromatin regions [155], and by altering chromatin repositioning. Since knockdown of *MALAT1* causes differential expression of several genes that localize away from the *MALAT1* locus [79], and because *MALAT1*-associated epigenetic-regulation of genes was found to happen exclusively in cis [159], it is tempting to speculate that the nuclear architecture is reorganized as a direct consequence of speckle assembly. Therefore, the assembly of subnuclear compartments might constrain chromatin into new locations in the 3D space, thereby affecting gene expression. Accordingly, knockdown of *NEAT1* impairs paraspeckles’ assembly [103] and also affects gene expression [80]. In fact, insights from studies on another lncRNA, *FIRRE*, show that it forms punctate compartments in the nucleus that include not only its own locus, but also specific loci from several other chromosomes [160]. This finding raises the question of whether SCS lncRNAs have the ability to co-localize to specific genomic regions in close proximity with its subnuclear compartments. A recent study also showed that SCS lncRNAs can interact with molecules that are present in the promoters of genes and remodel their chromatin by repositioning the loci into actively-transcribed or repressed foci [161]. Another question is whether age-related disturbances and the abundance of SCS lncRNAs impact the location and assembly of subnuclear compartments, consequently dictating broader rearrangements in 3D chromatin organization and altered gene expression patterns.
(3) An emerging view is that the act of lncRNA transcription possibly defines or affects the nuclear architecture [156]. This model suggests that the transcription of lncRNAs serves as a guide-post for shaping 3D genome organization and that, for this same reason, lncRNAs have low abundance and are tissue-specific [156]. It is plausible that qualitative and quantitative changes in lncRNA expression with aging can have major effects in the broad nuclear architecture of the cell and thus contribute to the loss of cellular identity.

It follows that long-term maintenance of the nuclear architecture by lncRNAs is vital for neuronal functioning, and that lncRNA-associated age-related disturbances have a broad impact on 3D nuclear architecture and may lead to neuronal dysfunction.

## 5. Perspective

In summary, we have discussed multiple examples whereby lncRNA activity is involved in aspects of neuronal function (Table 1), such as neurogenesis and synaptic function, and may allow for continuous reshaping of the nuclear architecture. In the context of aging, we also discuss how lncRNAs affect this genetic landscape through their involvement in transcriptional alterations of protein-coding genes, lncRNAs, and epigenetic alterations. Hence, the coding/non-coding interactome that sustains important processes of cognition and adult neurogenesis may become compromised during neuronal aging. It is not yet known whether changes in the transcription of lncRNAs are reactive, compensatory, or causative of aging. However, rapidly accumulating evidence supports the vital contribution of lncRNAs in neuronal aging.

## Figures and Tables

**Table 1:** Mechanisms underlying lncRNAs neuronal functions and their affected expression levels in aging and age-related neurodegenerative disorders.

Mechanisms Underlying lncRNA Activity			lncRNA	Implication in Aging/Age-Related Neurodegenerative Disorders	Affected Neuronal Process
Cytoplasm (post-transcriptional modulation of gene expression)	mRNA translation		*Bc1*, *BC200*	Downregulated in aging; upregulated in Alzheimer’s disease (AD) [86]	Cognitive decline
			*UCHL1-AS* [124]	Downregulated in Parkinson’s disease (PD) [127]	Neurodegeneration
	mRNA stability		*BACE1-AS* [121,122]	Upregulated in AD [122]	Protein aggregation in neurons; Possible role in cognitive decline
			*PINK1-AS* [131]	Unknown	Neurodegeneration
			*GDNF-AS* [77], *EPHB2-AS* [77], *CNA2-AS*	Unknown	Cognitive decline
	Sponge/decoy		*TUG1* [162]	Upregulated in human subventricular zone (SVZ) with aging [13]; upregulated in brain ischemia [163]	Adult neurogenesis decline; Possible role in cognitive decline and neurodegeneration
Nucleus (pre-transcriptional regulation of gene expression)	Transcription repression	by sequestration of chromatin-regulatory proteins	*LRP1-AS* [128]	Upregulated in AD [128]	Neurodegeneration [164]; Possible role in protein aggregation
		by affecting histone modifications	*BDNF-AS* [77]	Unknown	Cognitive decline; neurodegeneration
			*HOTAIRM*	Altered expression in all human tissues assayed in aging [138]; overexpressed in a mouse model of PD [139].	Possible role in neurodegeneration [139]
			*ANRIL*	Variants have been associated with AD [140]; altered expression in all human tissues assayed in aging [138].	Neurodegeneration
	Scaffold for proteins and RNAs in subnuclear compartments		*NEAT1*	Dysregulated expression in a temporal lobe epilepsy mouse model [80]; upregulated in the human SVZ with age [13]; upregulated in Frontotemporal Dementia (FTLD) and Amyotrophic Lateral Sclerosis (ALS) [165]; upregulated in the hippocampus of old mice [166].	Adult neurogenesis decline; cognitive decline
			*MALAT-1*	Upregulated in human SVZ with aging [13]; upregulated in FTLD-ALS [165]; upregulated in the hippocampus of old mice [167]; upregulated in PD mouse model [166].	Adult neurogenesis decline; cognitive decline; neurodegeneration
			*GOMAFU*	Upregulated in SVZ with aging [13]; downregulated in the grey matter of schizophrenia patients [72]; upregulated in the hippocampus of old mice [167].	Cognitive decline;
Unclear mechanisms			*Six3OS*, *Dlx1A-S*, *Lnc-OPC*	Unknown	Adult neurogenesis
			*LNC00657*	Downregulated in the human SVZ with age	Adult neurogenesis
			*Meg3*	Upregulated in the hippocampus of old mice [167]; downregulated in old induced striatal medium spiny neurons [168].	Cognitive decline
			*SORL1-AS* [123]	Upregulated in AD	Protein aggregation; Possible role in cognitive decline
			*17A* [169]	Upregulated in AD	Cognitive decline
